# Amazonian Odonata Trait Bank

**DOI:** 10.1002/ece3.10149

**Published:** 2023-06-15

**Authors:** Victor Rennan Santos Ferreira, Bethânia Oliveira de Resende, Rafael Costa Bastos, Joás Silva da Brito, Fernando Geraldo de Carvalho, Lenize Batista Calvão, José Max Barbosa Oliveira‐Junior, Ulisses Gaspar Neiss, Rhainer Ferreira, Leandro Juen

**Affiliations:** ^1^ Laboratório de Ecologia e Conservação (LABECO) Universidade Federal do Pará Belém Pará Brazil; ^2^ Laboratório de Estudos de Impacto Ambiental (LEIA), Instituto de Ciências e Tecnologia das Águas (ICTA) Universidade Federal do Oeste do Pará Santarém Pará Brazil; ^3^ Instituto Nacional de Pesquisas da Amazônia (INPA) Universidade Federal do Amazonas Manaus Amazonas Brazil; ^4^ Laboratório de Estudos Ecológicos em Etologia e Evolução (LESTES Lab) Universidade Federal do Triângulo Mineiro Uberaba Minas Gerais Brazil

**Keywords:** bibliography, database, functional traits, phenotype, raunkiæran shortfalls

## Abstract

Discussion regarding the gaps of knowledge on Odonata is common in the literature. Such gaps are even greater when dealing with basic biological data for biodiverse environments like the Amazon Rainforest. Therefore, studies that address, classify, and standardize functional traits allow the elaboration of a wide range of ecological and evolutionary hypotheses. Moreover, such endeavors aid conservation and management planning by providing a better understanding of which functional traits are filtered or favored under environmental changes. Here, our main goal was to produce a database with 68 functional traits of 218 Odonata species that occur in the Brazilian Amazon. We extracted data on behavior, habit/habitat (larvae and adults), thermoregulation, and geographic distribution from 419 literature sources classified into different research areas. Moreover, we measured 22 morphological traits of approximately 2500 adults and categorized species distributions based on approximately 40,000 geographic records for the Americas. As a result, we provided a functional matrix and identified different functional patterns for the Odonata suborders, as well as a strong relationship between the different trait categories. For this reason, we recommend the selection of key traits that represent a set of functional variables, reducing the sampling effort. In conclusion, we detect and discuss gaps in the literature and suggest research to be developed with the present Amazonian Odonata Trait Bank (AMO‐TB).

## Graphical abstract

We extracted AMO‐TB trait bank on Odonata's behavior, habit/habitat (larvae and adults), thermoregulation, and geographic distribution, from 419 literature sources. Moreover, we measured 22 morphological traits from ~2.500 adults and categorized the species distribution based on approximately 40.000 geographic records for the Americas. From this information, we produced a database with 68 functional traits of 218 Odonata species that occur in the Brazilian Amazon.

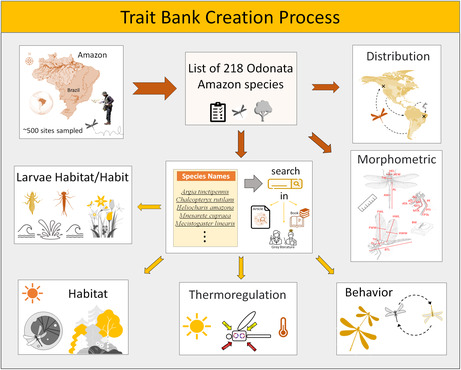

## 1 INTRODUCTION

The order Odonata is a group of insects that exhibit an amphibiotic life cycle, with an aquatic life stage as larvae that emerge as terrestrial adults (Suhling et al., 2015). They also have a long evolutionary history, with fossil records dating back to 268 million years ago in the Upper Permian (Bybee et al., 2021). Both features allowed colonization of diverse environments, such as forests, open fields, and water channels of different sizes, as well as several adaptations in morphology, behavior, and thermoregulatory traits, which consequently rule their life history and habitat selection (May, 1976).

Sexual maturity brings several changes, mainly those related to predatory and reproductive behaviors. Males that allocated their energy to prey capture during the larval stage, now adopt a reproductive life strategy as adults, with complex courtship displays (Gibbons & Pain, 1992; Guillermo‐Ferreira et al., 2015), territorial defense (Grether, 2019), and mate acquisition and guarding (Thornton & Switzer, 2015). Adult females need to develop skills to select the best mates (Pena‐Firme & Guillermo‐Ferreira, 2020) by identifying their ideal male patterns of color and behavior, as well as finding and choosing adequate oviposition sites (Guillermo‐Ferreira & Del‐Claro, 2011; Rodrigues et al., 2019).

Odonates mostly rely on environmental heat to perform their daily activities. Theoretically, morphological and behavioral patterns have a positive‐feedback relationship with heat gain and loss strategies. For instance, smaller species exhibit a larger surface volume ratio, hence, tend to thermoregulate by convection and depend strongly on environmental temperature (May, 1976). Conversely, larger species have a smaller surface/volume ratio and reduced heat loss by convection (De Marco et al., 2015), making them more tolerant to solar radiation and warmer temperatures (May, 1976). Endothermic species may not follow these patterns and generate heat by contracting wing muscles. Moreover, due to the specific thermoregulatory demands of adult odonates, anthropic changes to the landscape may cause species turnover from forest specialists to species that are open‐area specialists or habitat generalists (Calvão et al., 2018; Oliveira‐Junior & Juen, 2019; Mendoza‐Penagos et al., 2021).

The order Odonata is a small group when considering the biodiversity patterns observed in Insecta (May, 2019), with approximately 6303 species worldwide distributed among two suborders for the Neotropics – Zygoptera and Anisoptera (Bybee et al., 2021). These suborders reflect phylogenetic relationships, morphological, and physiological peculiarities (May, 2019). For example, Zygoptera species (damselflies) are usually smaller and slender, presenting lower dispersion capacity, and thermoregulate by heat exchange with the environment. These characteristics make them more sensitive to environmental changes (Oliveira‐Junior & Juen, 2019). On the contrary, Anisoptera species (dragonflies) are larger and more robust, with large wings that sustain longer flights. They also use using solar radiation for thermoregulation and in some cases generate internal heat (Castillo‐Pérez, Suárez‐Tovar, et al., 2022).

Several regions detain a high diversity of dragonflies and damselflies. A great part can be found in forest remnants and other heterogeneous environments, rich in microhabitats and freshwater resources (Paulson, 2006). As for the Amazon Rainforest, recent estimates indicate that there are 503 species in the Brazilian territory only (Brasil et al., 2021). Nevertheless, it is worth highlighting that this number is probably underestimated given the gaps in collections throughout South America (Miguel et al., 2022).

Such information gaps on odonates are frequently discussed in the literature (Bastos et al., 2019; Brasil et al., 2021; Carvalho et al., 2022; Miguel, Calvão, et al., 2017; Miguel, Oliveira‐Junior, et al., 2017), especially regarding the tropics where most of world species diversity is concentrated and less research is conducted (Hortal et al., 2015; Laurance, 2007). The scarcity of fundamental biological data—for example, on geographic distribution, behavior, and thermoregulation–threatens global conservation efforts and limits the testing of ecological and evolutionary hypotheses. In this scenario, the need for studies that address, classify, and standardize basic biology data for species in the tropics is evident, hence, providing the basis for new lines of research that are currently impossible to carry out due to the before mentioned gaps. Here, we sought to fill some gaps by building a traits database. Therefore, our objective was to present and describe all the steps for the elaboration and construction of the Amazonian Odonata Trait Bank: (i) measures of different body structures; (ii) a compilation of literature data on reproductive behavior, thermoregulation, and habitat/habits for both life stages (larvae and adults); and (iii) the compilation and curation of geographic coordinates to classify the distribution of Odonata recorded for the Brazilian Amazon. We also aim to detect and discuss gaps in the literature and suggest research to be developed with the present Trait Bank.

## 2 MATERIALS AND METHODS

### 2.1 Study area

The Amazon is one of the most extensive forests in the tropical zone, covering approximately 5.5 million km^2^, with 60% of this area located in Brazilian territory (Macedo & Castello, 2015). According to estimates, it has the greatest diversity of living organisms on Earth (Hoorn, 2010). Despite such biodiversity, this ecosystem has been suffering heavily from deforestation, mainly due to soy and livestock production (Nepstad et al., 2006), habitat fragmentation and fires (Alencar et al., 2015), as well as changes in aquatic environments by hydroelectric plants and mining (Coelho & Monteiro, 2007). The region has a humid tropical climate (code “Af”) according to the Köppen classification and the predominant vegetation is categorized as Rain Forest (Veloso et al., 1991), with portions of Amazonian savannas (local name “canga”) found in rocky outcrops (Souza‐Filho et al., 2019).

### 2.2 Trait Bank creation process

**FIGURE 1:**
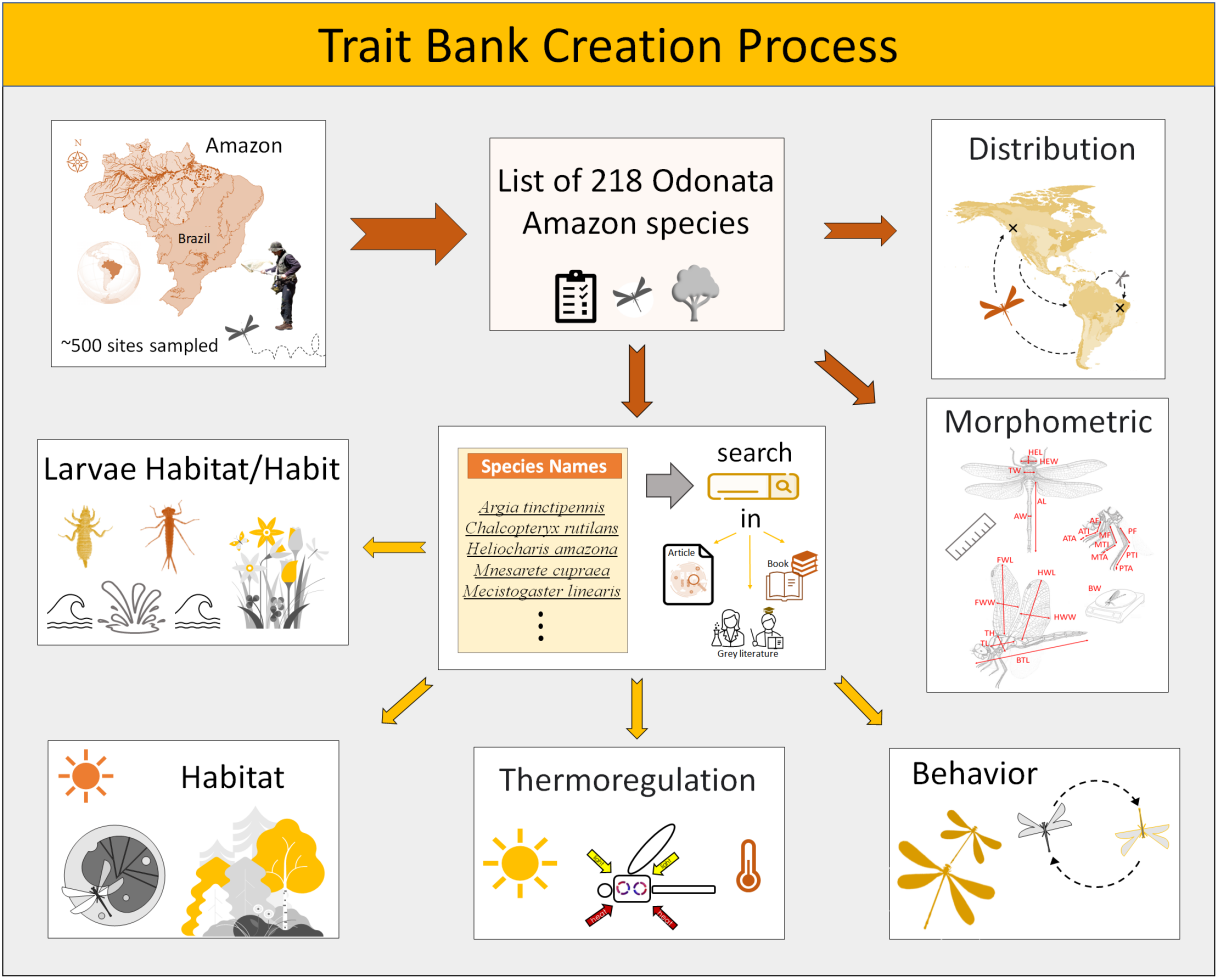
AMO‐TB development step by step. We gathered information from 419 literatures on 68 traits for 218 species of Odonata Amazon. Moreover, we measured 22 morphological traits and categorized the species distribution based on approximately 40,000 geographic records for the Americas. Available in: Dryad repository (https://doi.org/10.5061/dryad.brv15dvdg).

First, we organized a checklist of 218 species occurring in the Brazilian Amazon based on approximately 500 collection sites sampled over a decade by researchers of the Laboratory of Ecology and Conservation (LABECO) at the Federal University of Pará (see more in Mendoza‐Penagos et al., 2022). Subsequently, we started the selection of traits and their further categorization. We emphasize that some groups of traits present different life history facets, in this way justifying the classification in different groups. Therefore, a portion of the groups of the traits was created based on the “Odonate Phenotypic DataBase,” which presents a continuous survey of Odonata phenotypical data (Waller et al., 2019). Unfortunately, it depicts only a little information on neotropical species. Therefore, to build other groups of traits, we used specific scientific papers that provided larvae lifestyle information (Carvalho & Nessimian, 1998), thermoregulation aspects (Corbet & May, 2008; May, 1976, 1991), sexual behavior (Resende et al., 2021; Rodrigues et al., 2019), morphometrics (Pereira et al., 2019), and geographical distribution (Renner et al., 2019). Afterward, we carried out an active search for information on behavior, thermoregulation, geographic distribution, and habitats/habits of both life stages (larval and adult; see more in Table 1) in the scientific literature (books and articles) available in the databases: “Web of Science” and “Google Scholar” (literature available in Appendix 1). The search was performed using the respective names of the 218 registered species as keywords (species names available in Appendix 2—AMO‐TB). Due to the lack of published information for many species in the region, we complemented some information gaps by consulting specialists, as well as in “gray” literature such as theses and dissertations (Figure 1). Finally, despite all efforts, some species (especially the rarest or recently described) lack certain basic information. Therefore, we performed congener extrapolations for the categories of behaviors and habits/habitats of larvae and adults by detecting the most frequent trait within the genus.

**TABLE 1:** Classification and definition of the 68 traits compiled and measured for odonates of the Amazon Rainforest.

Traits group	Trait	Definition
Morphometric	Body total length	Distance between the distal tip of the head and the anal appendices
	Fore wing length	Distance from the base of the forewing to its apex
	Fore wing width	Middle forewing width
	Hind wing length	Distance from the base of the hindwing to its apex
	Hind wing width	Middle hindwing width
	Abdomen length	Distance between the proximal limit of abdominal S1 and the distal tip of the cercus
	Abdomen width	Middle abdomen width
	Thorax length	Distance between the mesepisternum proximal margin and the distal end of the metepimeron
	Thorax width	Distance between the thoracic ends in a dorsoventral plane
	Thorax height	Distance between the mesepisternum distal tip and the proximal end of the metepimeron
	Head length	Distance between clypeus distal apex and proximal limits of the eye
	Head width	Distance between the head tips in a dorsoventral plane
	Anterior femur	Distance between the proximal/distal tips of the anterior femur
	Anterior tibia	Distance between the proximal/distal tips of the anterior tibia
	Tarsus anterior	Distance between the proximal/distal tips of the anterior tarsus
	Medial femur	Distance between the proximal/distal tips of the medial femur
	Medial tibia	Distance between the proximal/distal tips of the medial tibia
	Medial tarsus	Distance between the proximal/distal tips of the medial tarsus
	Posterior femur	Distance between the proximal/distal tips of the posterior femur
	Posterior tibia	Distance between the proximal/distal tips of the posterior tibia
	Posterior tarsus	Distance between the proximal/distal tips of the posterior tarsus
	Body weight	Dehydrated body weight in (g)
Behavior	Territoriality	Copulation and oviposition in fixed territories (binary trait: yes/no)
	Tandem	The male holds the female after mating
	Non‐contact guarding	Male guards female but no physical contact
	No guard	Female alone, without the guard in oviposition
	Courtship	Exhibition for the female in the pre‐copulation stage (yes/no)
	Agonistic display	Resolve disputes without contact, using some type of display (yes/no)
	Endophytic	Lay eggs within living plant tissue
	Epiphytic	Oviposition on the exposed surface
	Exophytic	Lay eggs directly in the water
	Wood	Wood decomposition substrate
	Vegetal tissue	Plants, roots, mass algae, and debris underwater
	Water	Water surface
	Phytotelma	Water accumulated in the leaf sheaths of bromeliads and tree hollows
Adult Habitat Preference	Pond	A small amount of backwater, with intermittent duration
	Lake	A medium or large amount of dammed water, with perennial duration
	Marsh	Wetland dominated by grassy herbaceous vegetation, with ample solar incidence
	Swamp	Shaded wetland due to abundance of woody vegetation
	Stream	Water flowing within a channel
	River	Large running water system
	Forest	An area dominated by trees
	Open field	Open areas predominated by herbaceous vegetation (e.g., pasture and savanna)
Thermoregulation	Thermal conformer	Convective heat exchanges with the environment
	Heliothermic	Heat gain via direct solar radiation on the body
	Endothermic	Produces and distributes internal heat
	Percher	Sustains short flights and perched
	Flier	Fly constantly
Geographic Distribution	Amazon	Distribution restricted to the Amazon
	Narrow	Occurs in 2 biomes
	Dispersed	3–4 biomes
	Wide	5–6 biomes
	Very wide	More than 7 biomes
Larval Habitat Preference	Lentic	Closed water system, no water flow
	Lotic	Interconnected water system with current flow
	Inorganic sediment	Small matter originating from sedimentary processes (e.g., sand)
	Leaf litter	Organic matter composed of decaying leaves
	Debris	Large debris from branches and rocks
	Macrophytes	Plants that inhabit aquatic environments (e.g., *eichhornia* and *pontederia*)
	Roots	Root complex
	Phytotelma	Water accumulated in vegetable cavities
Larval Habits	Climber	Climbs branches and stems—vertical locomotion
	Swimmer	Swims by serpentine movements
	Clinger	Common in running waters, as they live clinging to debris, plants, and rocks
	Sprawler	Move over fine or floating sediments
	Burrower	Often buried in sedimented and inorganic substrate

We measured 22 adult morphological traits (detailed in Table 1 and Appendix 3) with the aid of a stereomicroscope, a digital caliper (precision of 0.01 mm), and an analytical scale (precision of 0.0001 g). Additionally, to optimize the accuracy of these measurements, as well as to dilute possible natural intraspecific variance, we repeated the measurement of each of the structures three times in at least five male specimens of each species. Finally, from the average of these measurements, we generated a fixed value of each trait for the species included in the study.

Finally, we surveyed the occurrence records of the 218 studied Amazonian species for the entire American continent. Part of these records was obtained from the digital platforms “species link” (http://splink.cria.org.br/) and “gbif” (https://www.gbif.org/). In addition, data provided/shared by different Brazilian research groups (LABECO, LENX, LESTES Lab) were included, as well as records available in more than 2400 articles published for South America. All the data compiled at this stage added up to more than 40,000 occurrence records for the Americas (bibliography and records available in Appendix 4—AMO‐TB). Subsequently, each of these species was classified according to its distribution amplitude along the biomes of the American continent (Olson et al., 2001, Appendix 5—Figure): (i) short distribution (2 biomes); (ii) dispersed distribution (3–4 biomes); (iii) wide (5–6); and (iv) very wide (>7) distribution. The data processing and species classification processes were performed using the R environment (Development Core Team, 2022) (script available in Appendix 6). It is worth mentioning that all species classified as “distribution restricted to the Amazon” were validated from distribution maps prepared by the International Union for Conservation of Nature (IUCN,  2022).

The AMO‐TB includes several metadata such as bibliographic reference files, figures, geographic coordinates, and scripts. We provide these appendices (1–9) in the online repository Dryad (https://doi.org/10.5061/dryad.brv15dvdg).

### 2.3 Dispersion and similarity of traits

To visualize possible patterns of species distribution in multidimensional space based on multiple traits, we used a principal coordinates analysis (PcoA) (Gower, 1966). This analysis was carried out separately by trait group once those categories contemplate different aspects of the life history of Odonata species. The appropriate methods of data transformation and similarity matrices were applied to each PcoA due to the plurality of data (continuous and/or discrete). Moreover, we used the fuzzy coding system to codify the binary data (adult habitat and habits/habitats of larvae). This procedure allows us to incorporate weighted information over habitat and habits plasticity from different records of the same species. Therefore, our final variable is an affinity index, whose values range from zero (no affinity for a particular category) to 100 (high affinity; Chevenet et al., 1994). We used the generalized Gower method with fuzzy variables and binary data distribution (Pavoine et al., 2009). For continuous data, we used standardization and Euclidean distance methods. We did not sort the thermoregulation data due to the great lack of information within this category (see more in Results and Discussion). In addition, the ordinations were made separately by suborder, due to the biological and ecophysiological distinctions of the groups. It is worth noting that in the plots, we also highlighted genera with different colors, to indicate that the taxonomic closeness among scores (representing species) is also explained through phylogenetic relationships. Finally, we performed a Pearson correlogram to demonstrate which morphometric traits are highly correlated, thus indicating a subset of variables that should be prioritized for future studies with odonates.

## 3 RESULTS

We record and measure a total of 68 different traits for 218 odonate species widely distributed in the Amazon rainforest. These species are distributed in 58 genera and nine families. We summarize the results from AMO‐TB and discuss potential implications.

### 3.1 Morphometrics

We selected 22 adult morphological traits that reflect basic requirements for odonate survival. These traits varied from small structures (leg parts) to the length/width of body parts (i.e., head, thorax, abdomen, and wings). Data were gathered from measurements of ~2500 individuals and 240 morphometrical records from the literature (available in Appendix 7—AMO‐TB).

**FIGURE 2:**
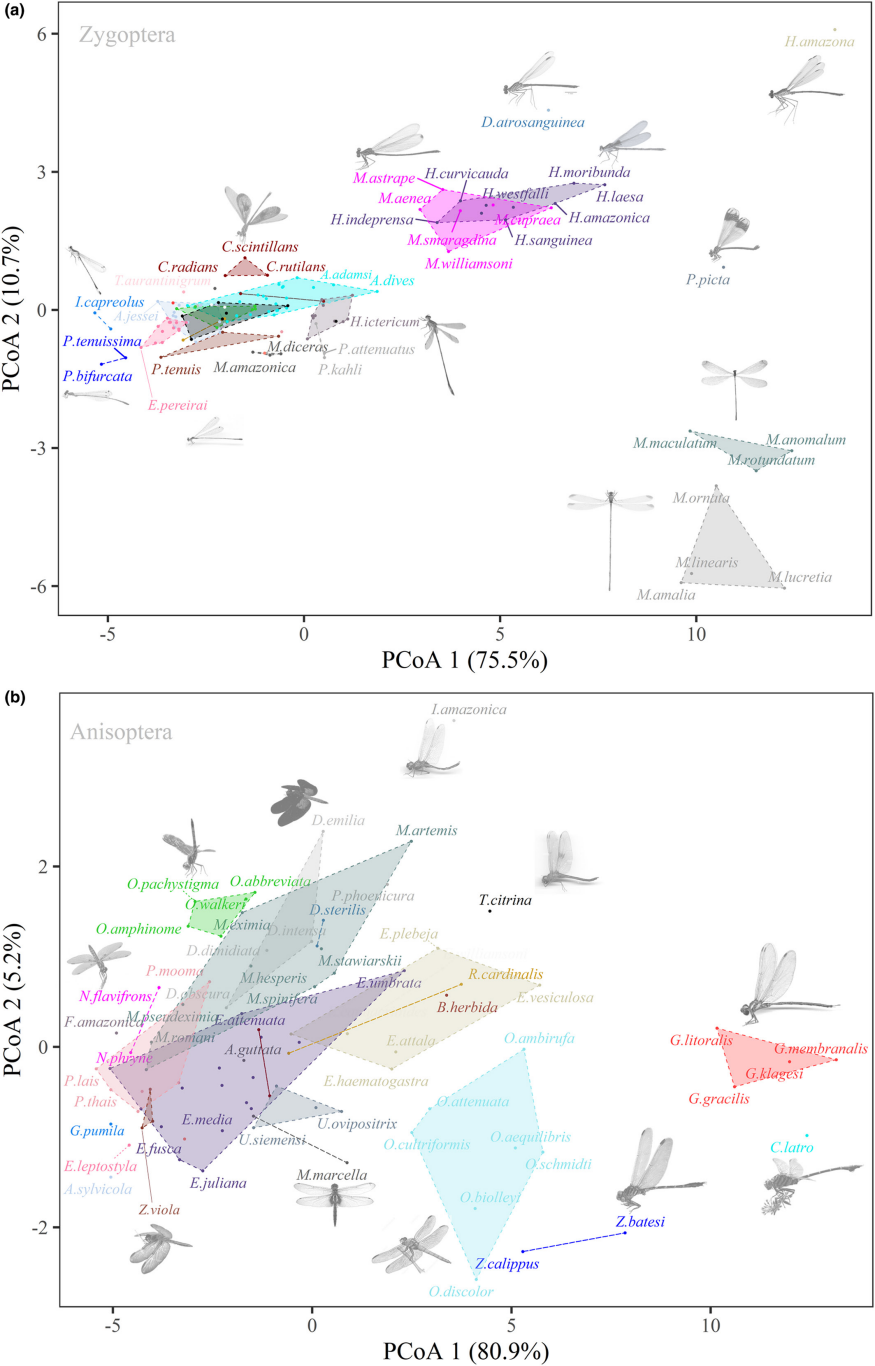
Ordering of species according to 22 morphological traits of the different Odonata suborders. The colors represent the different genera.

Results of the morphological space of damselflies represented by the ordination of the PcoA (Figure 2a) show that *Mecistogaster* (Rambur, 1842) species exhibit the larger body sizes and wing lengths of forewings (FW), seconded by the dicteriadids *Heliocharis amazona* (Selys, 1853) and *Dicterias atrosanguinea* (Selys 1853), which also exhibit the longer legs among damselflies, and the calopterygids *Hetaerina* (Selys, 1853) and *Mnesarete* (Cowley, 1934). *Ischnura capreolus* (Hagen, 1861) and *Ischnura fluviatilis* (Selys, 1876) have the lowest values of weight, wing length, thorax width, and some leg measurements. The polythorids, *Chalcopteryx* (Selys, 1853) and *Polythore* (Calvert, 1917), remained isolated from other zygopteran species due to their peculiar morphology, with asymmetrical fore and hindwings, broad abdomen, and quadrangular thorax (Figure 2a).

Analyzing the representatives of Anisoptera (Figure 2b), morphometrics such as thorax size, width of wings, and head were highlighted for *Cacoides latro* (Erichson, 1848), as well as dragonflies classified as fliers and, therefore, with well‐developed wing muscles, such as *Diaphlebia angustipennis* (Selys, 1854) and *Gynacantha* (Rambur, 1842), all belonging to families of mostly endothermic species, that is, Gomphidae and Aeshnidae. On the contrary, the species of the genus *Perithemis* (Hagen, 1861) (Libellulidae) stood out as the smallest Amazonian dragonfly. However, it is worth mentioning that, although these small dragonflies stand out for their smaller body size, they do not necessarily present the smallest sizes in the other variables. On the contrary, the genus *Orthemis* (Libellulidae) is represented by relatively large species.

**FIGURE 3:**
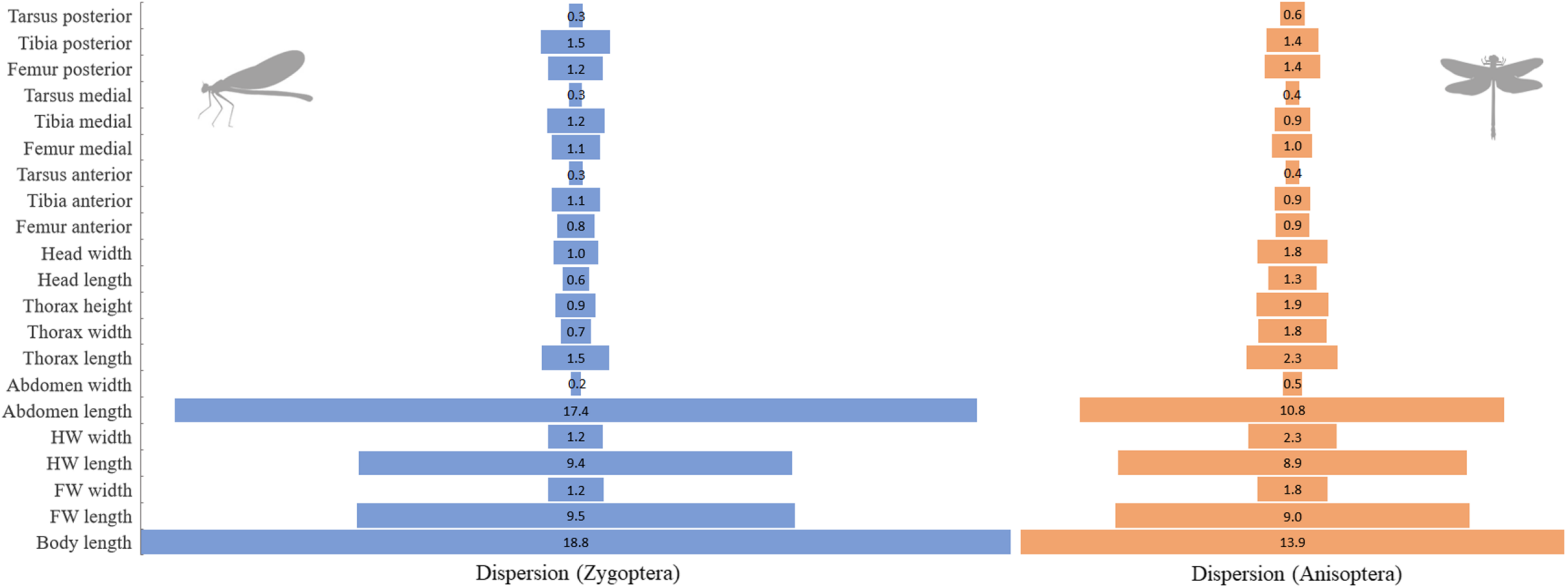
Morphological standard deviation found in different Odonata suborders occurring in Amazon Rainforest. HW, Hind Wing and FW, Fore Wing.

Regarding the ordination of suborders, it is noteworthy that there is a major variation in the morphometrics of dragonflies, as well as among genera. For example, the genera with the highest numbers of species (e.g., *Erythrodiplax*, *Micrathyria*, and *Erythemis*) show wide divergence in their measurements. Meanwhile, damselflies genera such as *Argia* (Rambur, 1842), *Epipleoneura* (Williamson, 1915), and *Acanthagrion* (Selys, 1876) occupied small spaces of morphometric variation in the ordering. Furthermore, it is evident that the total body size and length of the abdomen and the length of the wings vary greatly within both suborders when observing the standard deviation of the morphometric traits (Figure 3). This variation is greater in Zygoptera than Anisoptera, largely due to the peculiar shape of Pseudostigmatidae *stricto* sensu. However, there are similarities between the patterns of deviation between the suborders, mainly concerning the leg segments (femur, tibia, and tarsus).

**FIGURE 4:**
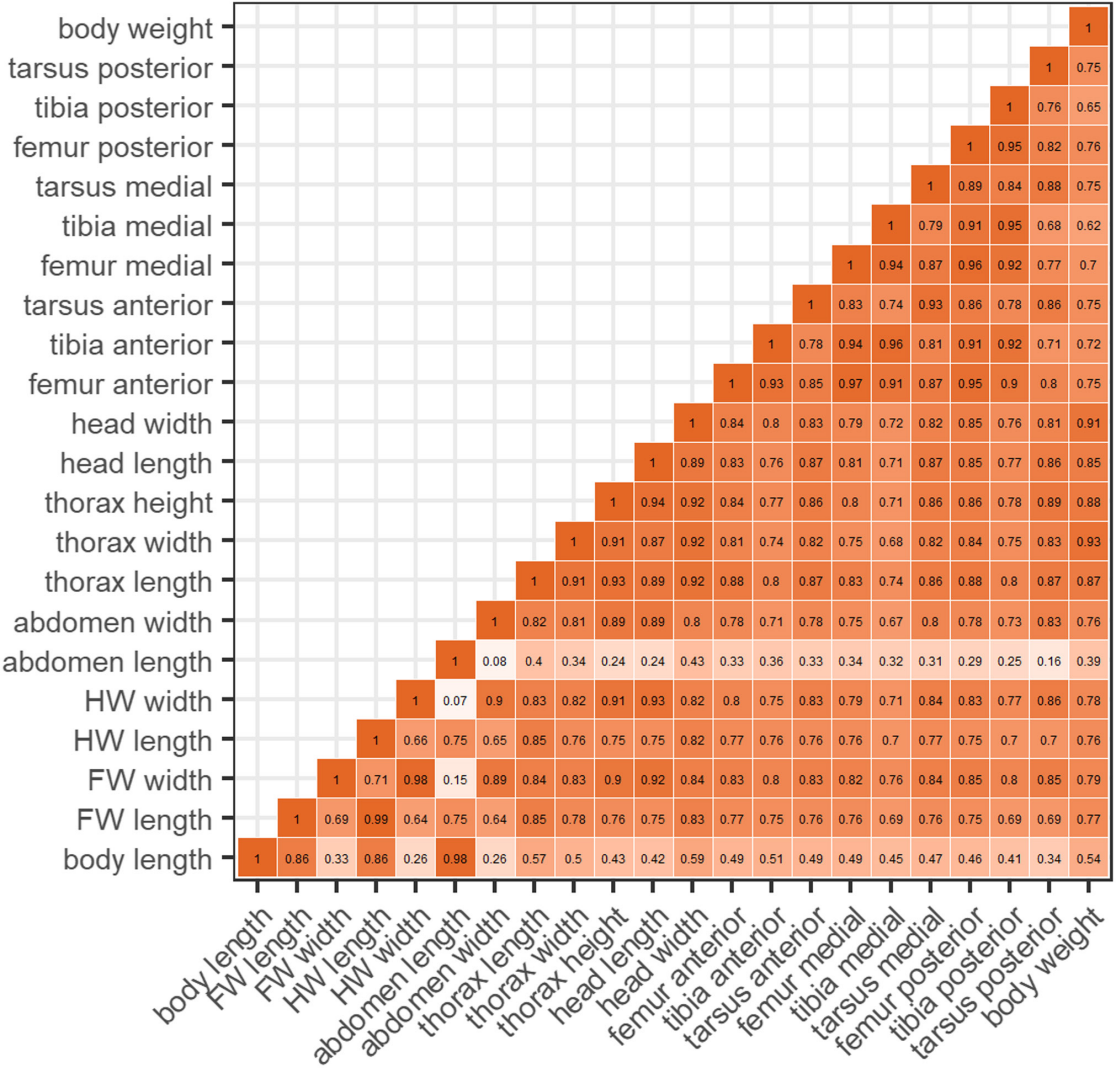
Correlogram (Pearson method) of the 22 morphological traits of Odonata occurring in the Amazon. FW = Fore Wing and HW = Hind Wing. The color intensity indicates a coefficient gradient. Orange = Higher Ratio and Salmon/White = Lower Ratio.

When evaluating the morphological correlation matrix (Figure 4) for the order Odonata, some patterns and conclusions appeared. For example, the length and width of the same wing do not exhibit a high correlation percentage (69%). On the contrary, the shape of the fore and hind wings is generally very similar between species. Moreover, there is a minimal correlation between the length and width of the abdomen (8%). Conversely, the length, width, and height of the thorax are correlated, with a correlation percentage of 94%, 92%, and 84%, respectively. Furthermore, the length and width of the head present a strong correlation (89%), with either measurement being a valid representation of head dimensions. We recommend the width, as it is easier to measure. Additionally, the leg segments are isometric, with minor exceptions for the correlations between tibia and tarsus sizes. The body weight tends to correlate with other measurements, especially thorax and head measurements. However, there are exceptions to this pattern due to non‐collinearity of weight, abdomen length, and total body size. Lastly, the total body size and the length of the abdomen showed low correlation values with most other morphometric traits, hence, important traits to be maintained in studies on morphology.

### 3.2 Reproductive behaviors

**FIGURE 5:**
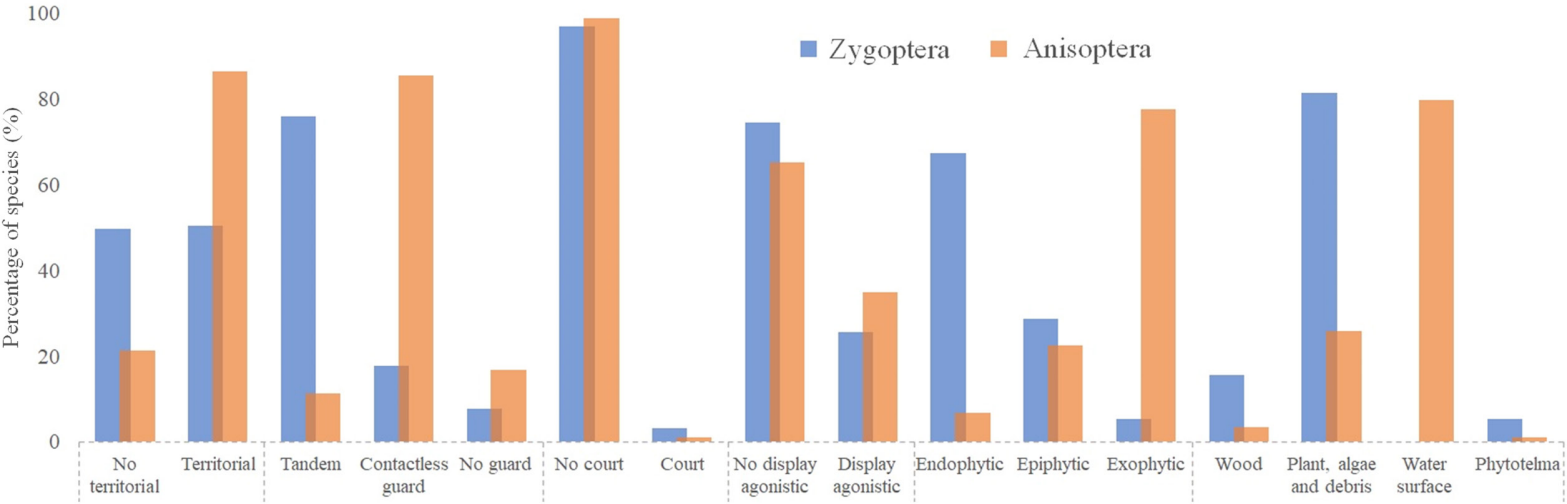
Proportion (%) of Odonata species present in the Amazon according to their reproductive behavior. The colors of the bars indicate the suborders.

The most frequent behavior was territoriality (yes = 63% X no = 37%), with nine species classified with ambiguity, that is, reported as both territorial and non‐territorial. The same pattern is repeated when restricting this analysis to Anisoptera, with a higher proportion of species that defend fixed territories (yes = 81% X no = 19%). However, this territorial dominance was not registered for Zygoptera, as there was a balanced proportionality in the presence and absence of this behavior (yes = 50% X no = 50%; Figure 5).

When we considered the presence of agonistic displays, most of the analyzed species did not show agonistic displays at the time of the dispute. When comparing the frequencies of these traits between suborders, Anisoptera has a higher proportion of species that exhibit contest behaviors, which is consistent with the previous result of greater territoriality for this suborder. Regarding courtship behavior, males of most Amazonian species do not perform any type of courtship displays to females (yes = 97% X no = 3%; Figure 5). Only six species with some type of pre‐mating courtship were recorded, almost all polythorids (see more in Appendix 2—AMO‐TB). It is worth mentioning that this result may be underestimated, due to the scarcity of literature that evaluates and discusses this behavioral trait (more detailed in the Literary Gap topic).

Within the mate‐guarding category, most Odonata species exhibit tandem behavior, followed by non‐contact guarding behavior. Additionally, some species overlap different traits within this category. When dealing with separate suborders, the tandem and non‐contact guard categories are inversely proportional between damselflies and dragonflies, with the absence of guarding behavior being uncommon within both suborders (Figure 5). Evaluating the types of oviposition, the studied dragonflies showed a higher frequency of endophytic behavior (41%), followed by exophytic (34%) and epiphytic (25%). We also observed an inverse pattern between the suborders, where Zygoptera presents more endophytic species (67%) while Anisoptera, is exophytic (78%). Additionally, Amazonian dragonflies mainly prefer plants as oviposition substrates (56%). When we analyzed the suborders, the pattern was maintained, given that for Anisoptera (mostly exophytic) the highest frequency of oviposition was on the water surface (80%) and for Zygoptera, it was live plant tissue (81%; Figure 5).

**FIGURE 6:**
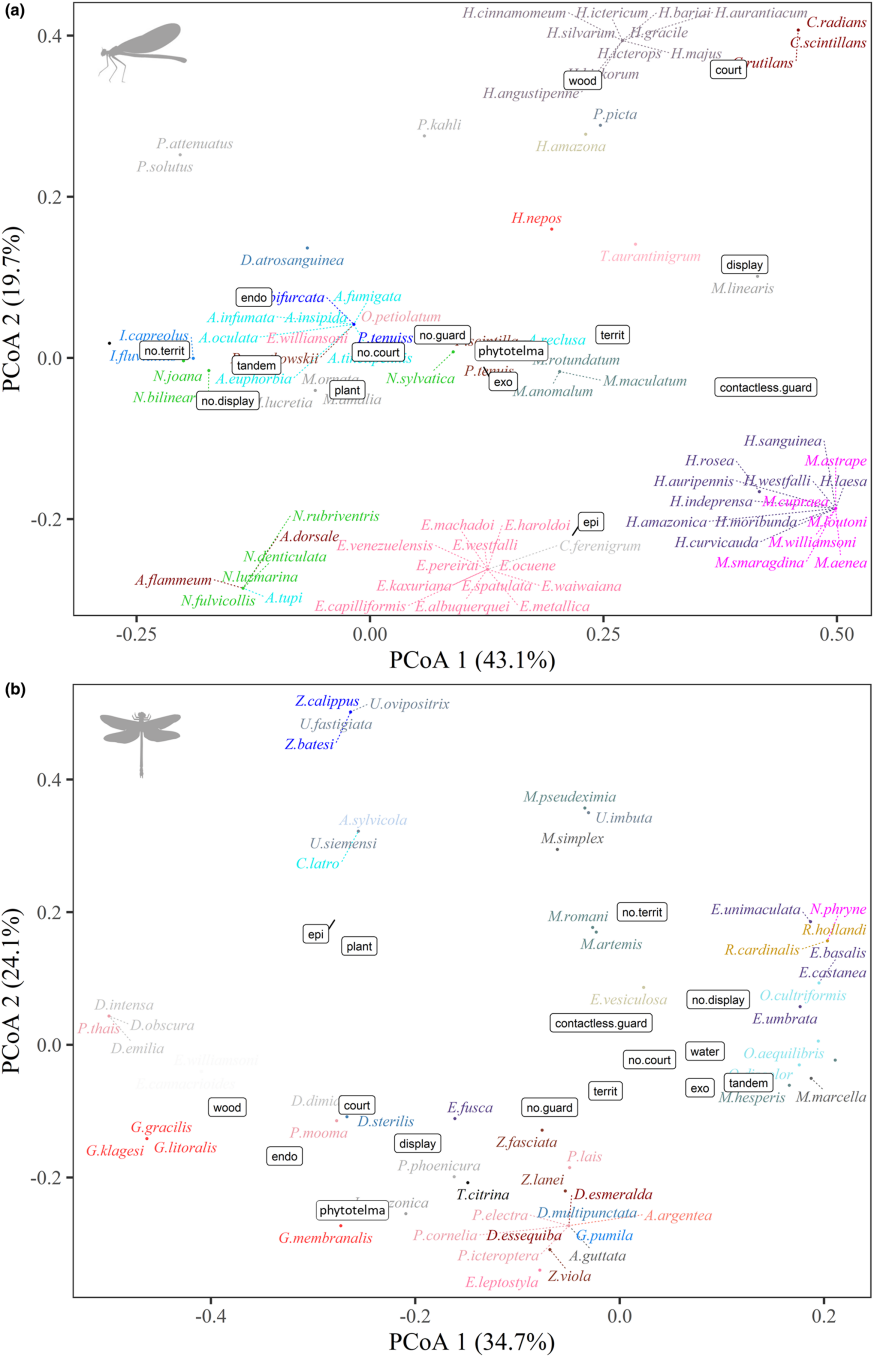
Ordering of species belonging to the two suborders of Odonata, (a) Zygoptera, (b) Anisoptera, according to their different reproductive behaviors adopted in nature. The colors categorize species belonging to the same genus.

In evaluating genera of damselflies, *Heteragrion* (Selys, 1862), *Polythore*, and *Chalcopteryx* showed a preference for decomposing wood for oviposition, with the difference that the last two genera are the only ones to present courtship behavior among the Amazonian species (Figure 6a). We also noted that the epiphytic behavior is homogeneous within the genera *Epipleoneura* (Williamson, 1915), *Mnesarete*, and *Hetaerina*. In addition, members of the Calopterygidae family are also extremely linked to female guarding behavior at a distance, a trait that is widely reported in the literature for this specific group. For *Argia*, the most diverse genus in the Neotropical region, the oviposition behavior of the endophytic type was the most reported one, that is, within living plant tissues (Appendix 2—AMO‐TB).

As demonstrated earlier, the frequency of behavioral traits within Odonata suborders is in many cases divergent. In Anisoptera, some species stand out with peculiar behaviors for the group, as is the case of the *Erythemis* (Hagen, 1861) species, in which oviposition occurs by the deposition of a mass of eggs that settle on roots, branches, and lianas along the channel's edge. The *Gynacantha* females lay their eggs inside micro grooves in wood made by their robust ovipositor, a common behavior in Aeshnidae, except for *Gynacantha membranalis* (Karsch, 1891), since their larvae were also found inside phytotelma. Additionally, the *Uracis* (Rambur, 1842) (Libellulidae), *Zonophora* (Selys, 1854), *Cacoides* (Cowley, 1934), and *Agriogomphus* (Selys, 1869) (Gomphidae) showed epiphytic behavior, generally opting for oviposition in plant debris and algae conglomerates (Figure 6b).

### 3.3 Adult habitat preference

**FIGURE 7:**
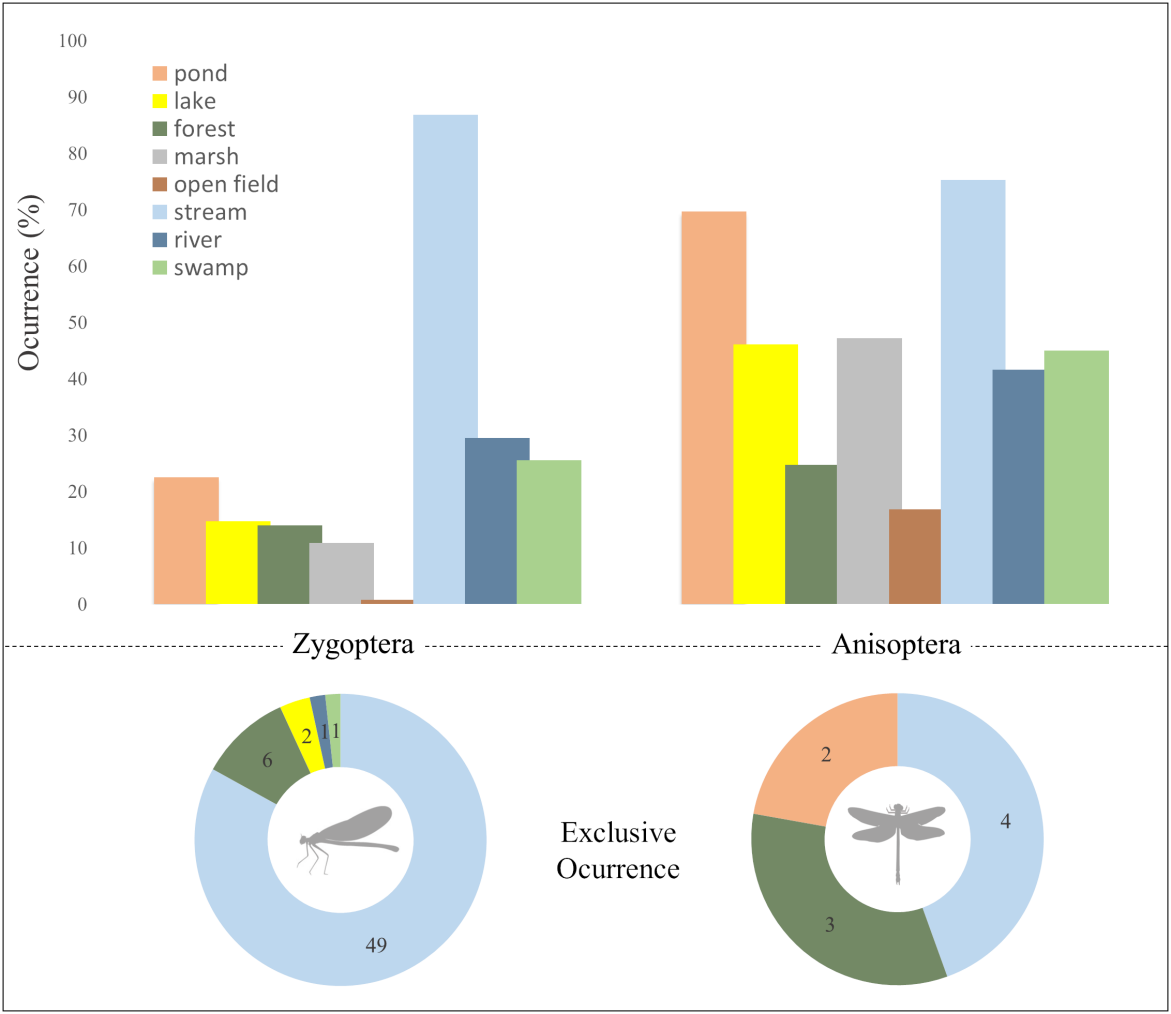
Frequency of occurrence (%) shared (upper) and number of exclusive species (lower) of Odonata suborders in different aquatic and semi‐aquatic habitats found in Amazonia. The colors represent different environments and those not illustrated in the pie graph show the absence of unique species (see more in Appendix 2—AMO‐TB available: Dryad repository).

The only dragonfly reported for all environments was *Erythrodiplax castanea* (Burmeister, 1839) (8/8), but it is also worth noting that eight species occurred in almost all environments (7/8), as is the case of *Miathyria marcella* (Selys, 1857), *Perithemis mooma* (Kirby, 1889), other *Erythrodiplax* species and even a small zygopteran *Ischnura capreolus*. All species are extremely abundant in the Americas (see more in the topic Geographical distribution). On the contrary, several species were restricted to only one type of environment, where the suborder Zygoptera has more exclusive species (*n* = 59/129) than Anisoptera (*n* = 9/89). In both cases, this restriction was more frequent to aquatic ecosystems of the stream type (Appendix 2—AMO‐TB) (Figure 7). Additionally, the species occurrence in forest and open field environments were the least recurrent in the literature.

**FIGURE 8:**
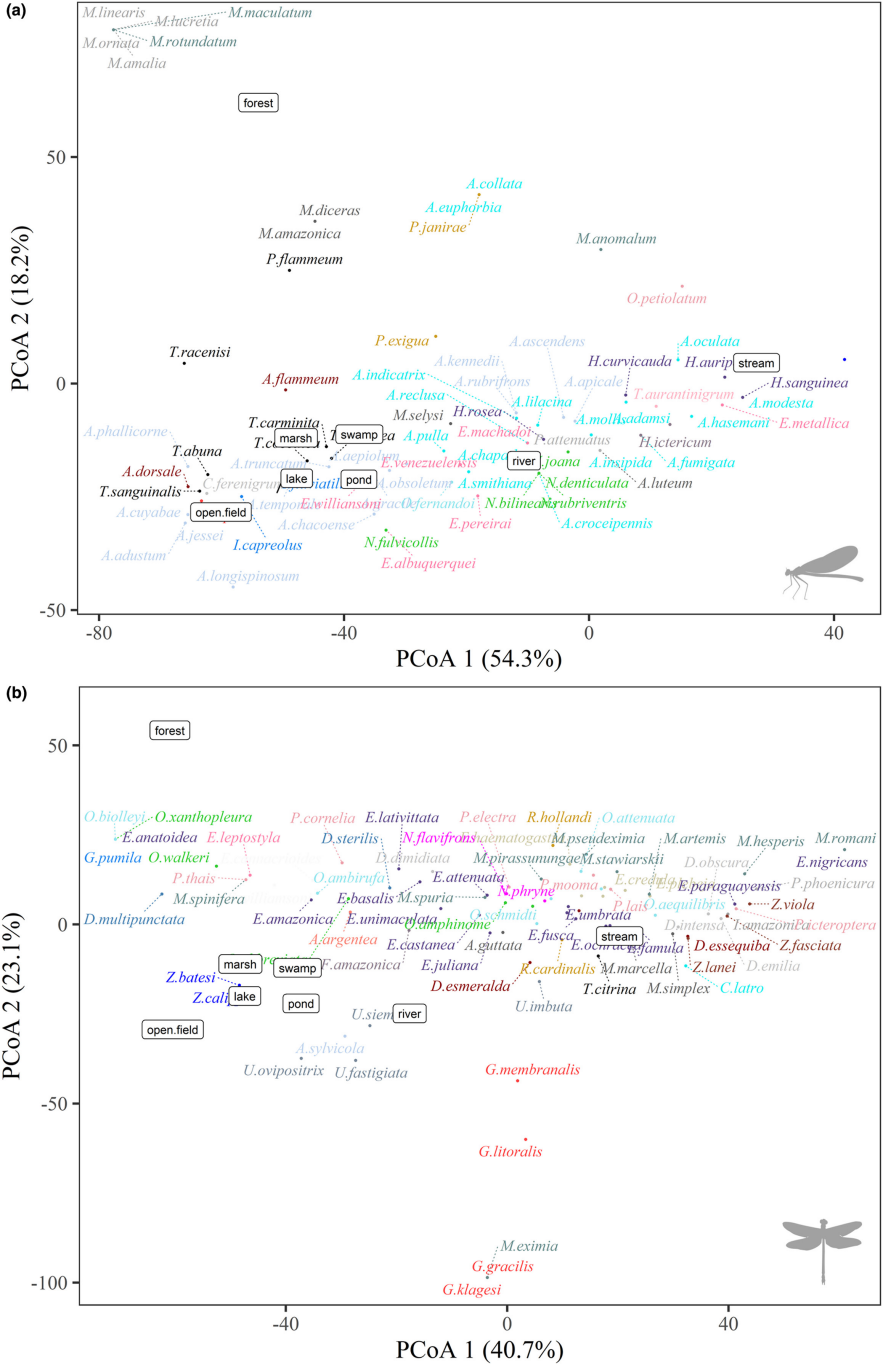
Ordering of species of the suborders of Odonata (a) Zygoptera, (b) Anisoptera, concerning the different habitats colonized by them. The colors categorize species belonging to the same genus.

When we analyzed the location of the ordered species based on their colonized habitats, we can observe that pseudostigmatids and dragonflies, such as *Orthemis* (Hagen, 1861), *Elasmothemis* (Westfall, 1988), and *Elga* (Ris, 1911), are directly related to forest environments (Figure 8a). In the other environmental categories, *Epipleoneura*, *Hetaerina*, and *Neoneura* species are often related to lotic environments (streams and rivers), while *Acanthagrion* (Selys, 1876), *Ischnura* (Charpentier, 1840), and *Telebasis* (Selys, 1865) to lentic environments. A similar pattern occurs for the genera of Anisoptera, with a clear distinction from the other environments (Figure 8b).

### 3.4 Thermoregulation

**FIGURE 9:**
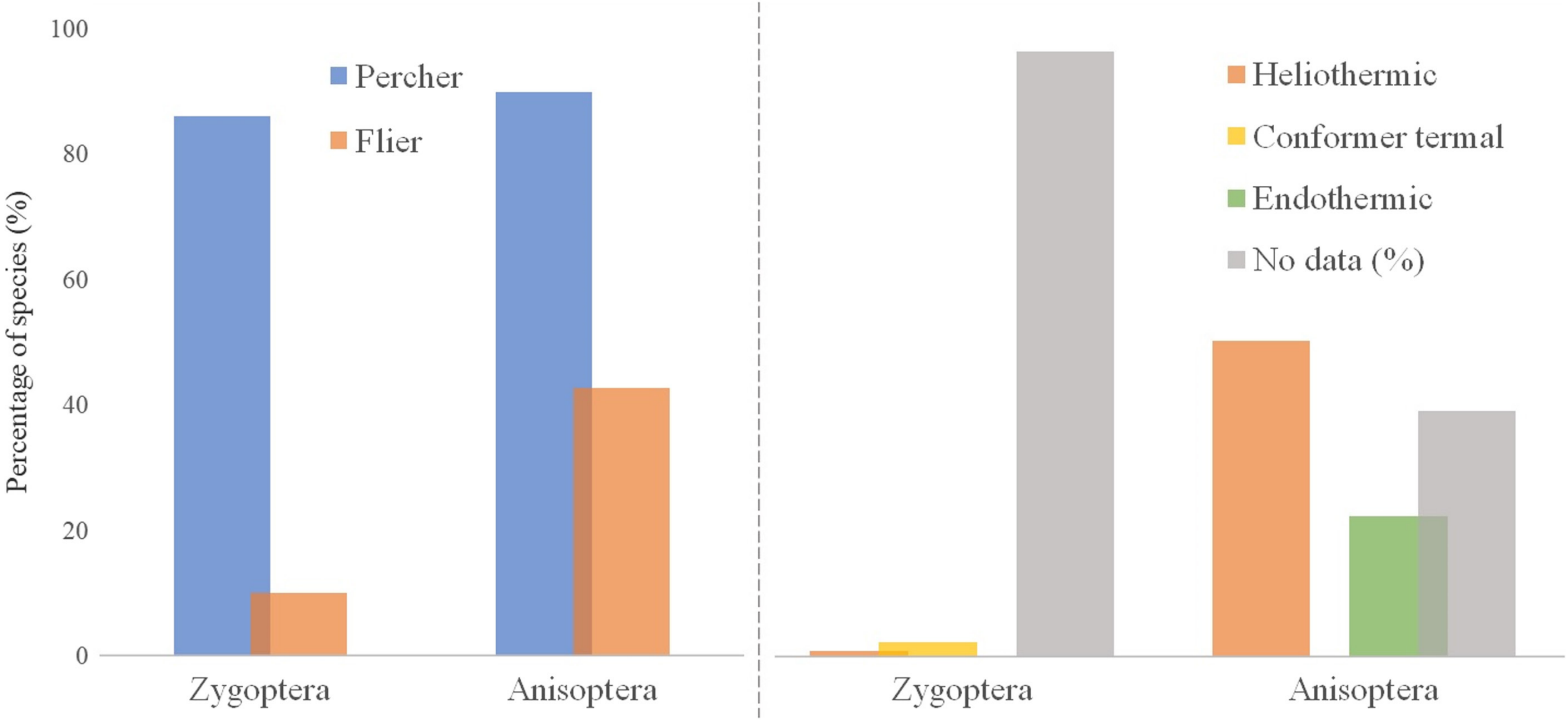
Flight and thermoregulation strategies adopted by the species of the different suborders of Odonata typical of the Amazon. The colors represent different categories.

When we analyzed both the order and the suborders of Odonata separately, most species are perchers. However, it is noteworthy that the number of flier species is substantially higher in Anisoptera than Zygoptera (Figure 9). As for the thermoregulatory strategies adopted by species, the information is so scarce that for Zygoptera we found data for only four species: *Aceratobasis macilenta* (Rambur, 1842), *I. capreolus*, *I. fluviatilis* (thermal conformers), and *Hetaerina rosea* (Selys, 1853) (heliothermic). For Anisoptera, we compile information for 54 species, 34 of which are categorized exclusively as heliothermic, nine solely as endothermic, and 11 classifieds with dualities between heliothermic and endothermic, often called “behavioral endotherms” (see more in Appendix 2).

### 3.5 Geographic distribution

**FIGURE 10:**
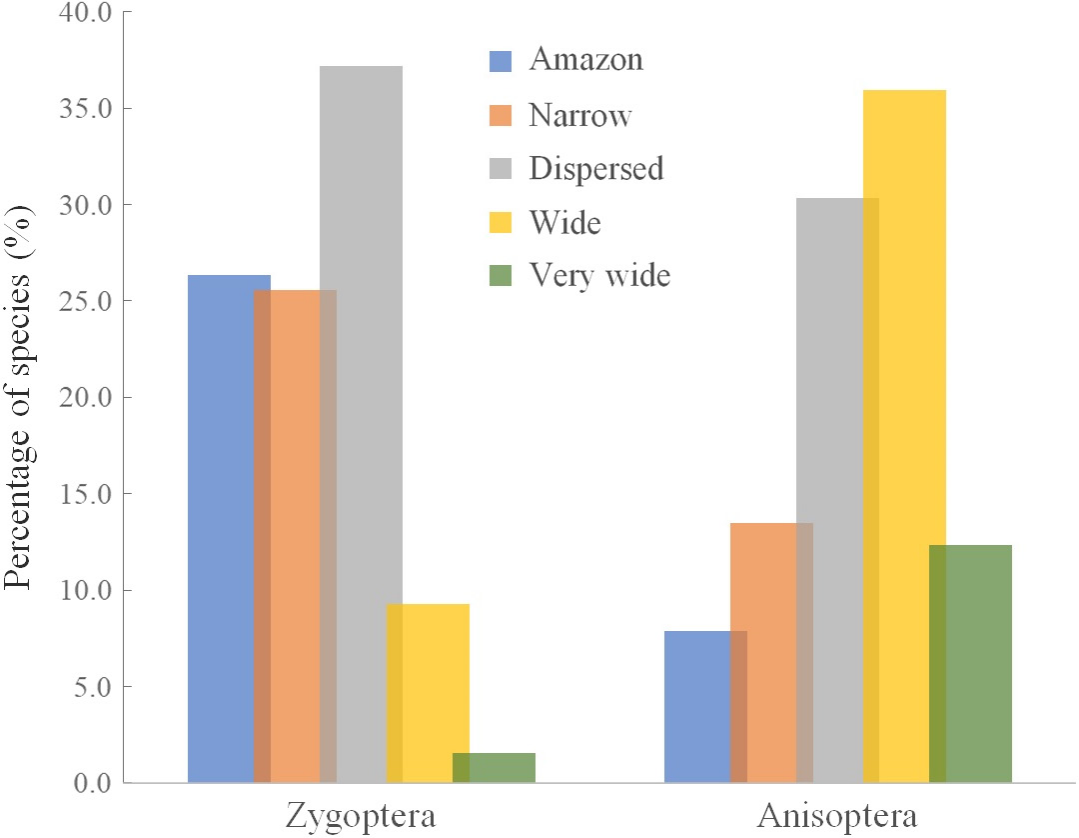
Classification of Odonata species occurring in the Amazon based on the number of biomes colonized by them. The colors reflect different distribution categories (detailed in Appendix 4 and 8—AMO‐TB available: Dryad repository).

For the 218 species of Odonata, we have compiled more than 40.000 occurrences recorded across the American biomes. Of these, 19% (41 spp.) are species restricted to tropical rainforest, 20% (44 spp.) have a short distribution, 35% (76 spp.) have a dispersed distribution, 20% (44 spp.) have a wide distribution, and 6% (13) very broad. Analyzing by suborders, we show that the classification patterns are inverted, while the Zygoptera presented a more restricted distribution to the Amazon (26%) and a short distribution (25%), the Anisoptera presented, for the same categories, respectively, 8% and 13% (Figure 10). On the contrary, we detected 36% of Anisoptera species with wide distribution and 12% very wide (all Libellulidae), against, respectively, 9% and only 2% for Zygoptera, with *Ischnura* being the only Zygoptera genus with distribution in more than seven biomes (see more in Appendix 4 and Appendix 8).

### 3.6 Larval habits and habitats

**FIGURE 11:**
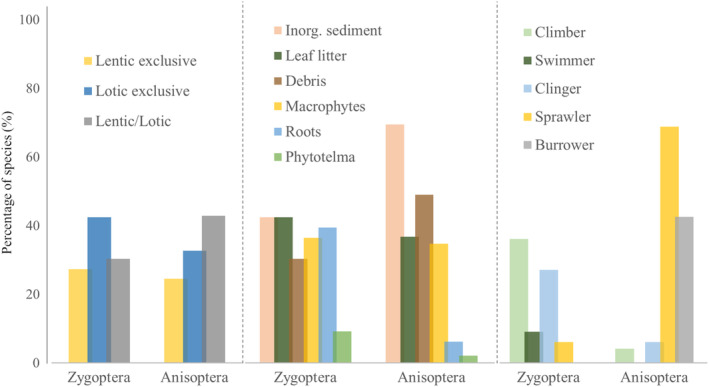
Distribution of genera (in %) of Odonata larvae typical of the Brazilian Amazon in relation to their habitats and adopted habits. The colors represent different categories of traits (detailed in Appendix 2—AMO‐TB available: Dryad repository).

Regarding the habitats of odonate larvae, most damselflies larvae occur in lotic environments (42%), while most dragonflies inhabit both lotic and lentic environments (43%). Considering the microhabitats, damselflies genera occur mainly in inorganic sediments (42%), litter (42%), and roots (39%), with only 9% of the genera in phytotelma. Most dragonfly genera also occur in inorganic sediments (69%), followed by fragments of branches, trunks, rocks (49%), and litter (37%). Furthermore, only *Gynacantha* occurs in phytotelma. Finally, regarding the larval habits, most zygopteran genera are climbers (36%) and clingers (27%), with no genus with burrowing habits. However, in Anisoptera, the most common type of habit among the genera are sprawlers (69%) and burrowing larvae (43%), with no larvae with swimming habits (Figure 11).

**FIGURE 12:**
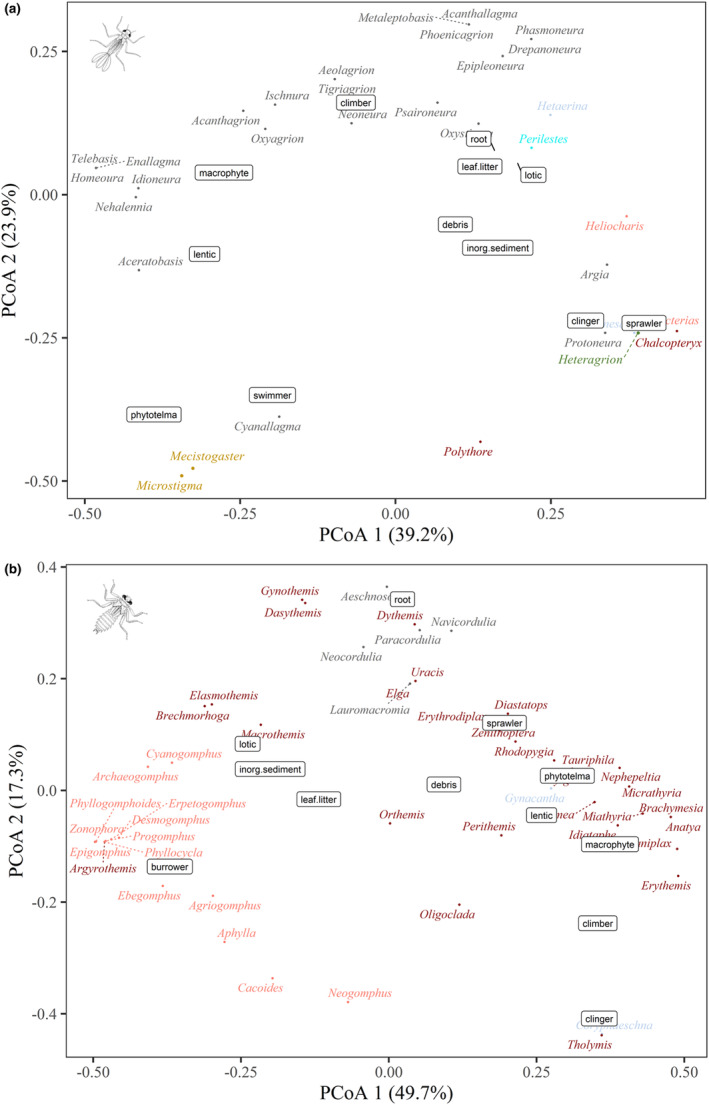
Ordering of the genera of the larvae of both suborders present in the Americas, (a) Zygoptera and (b) Anisoptera, concerning the types of aquatic environments colonized, substrates of preference, and types of habits. The colors categorize species belonging to the same family.

The zygopterans, *Argia*, *Heteragrion*, *Mnesarete*, *Dicterias* (Selys, 1853), *Protoneura* (Selys, 1857), *Chalcopteryx*, and *Heliocharis* (Selys, 1853) are related to the type of clasper habit, but only *Argia* and *Heteragrion* are also sprawlers. Analyzing the preference for microhabitat and water flow, *Aceratobasis* (Kennedy, 1920) (Coenagrionidae) and *Microstigma* (Rambur, 1842) and *Mecistogaster* (Rambur, 1842) (Pseudostigmatidae), are exclusive to phytotelma, usually with large amounts of water and organic matter. Within the Perilestidae, *Perilestes* (Hagen, 1862) was related to litter substrates and fragments of trunks, branches, and rocks in lotic environments. Considering the Anisoptera families, gomphids are related to burrowing habits, litter substrates, and inorganic sediments. While the Corduliidae is related to root substrates. In contrast to these specialist genera, the Libellulidae is widely distributed, occurring in a wide range of substrates, and presenting a relationship with various habits. We also highlight that *Tholymis* (Hagen, 1867) is exclusively a clinger, and *Coryphaeschna* (Williamson, 1903) is a clinger/climber. Regarding the type of substrate, *Gynacantha* is the only genus of the Anisoptera studied here that has a representative associated with phytotelma (Figure 12).

### 3.7 Gaps in the literature

**FIGURE 13:**
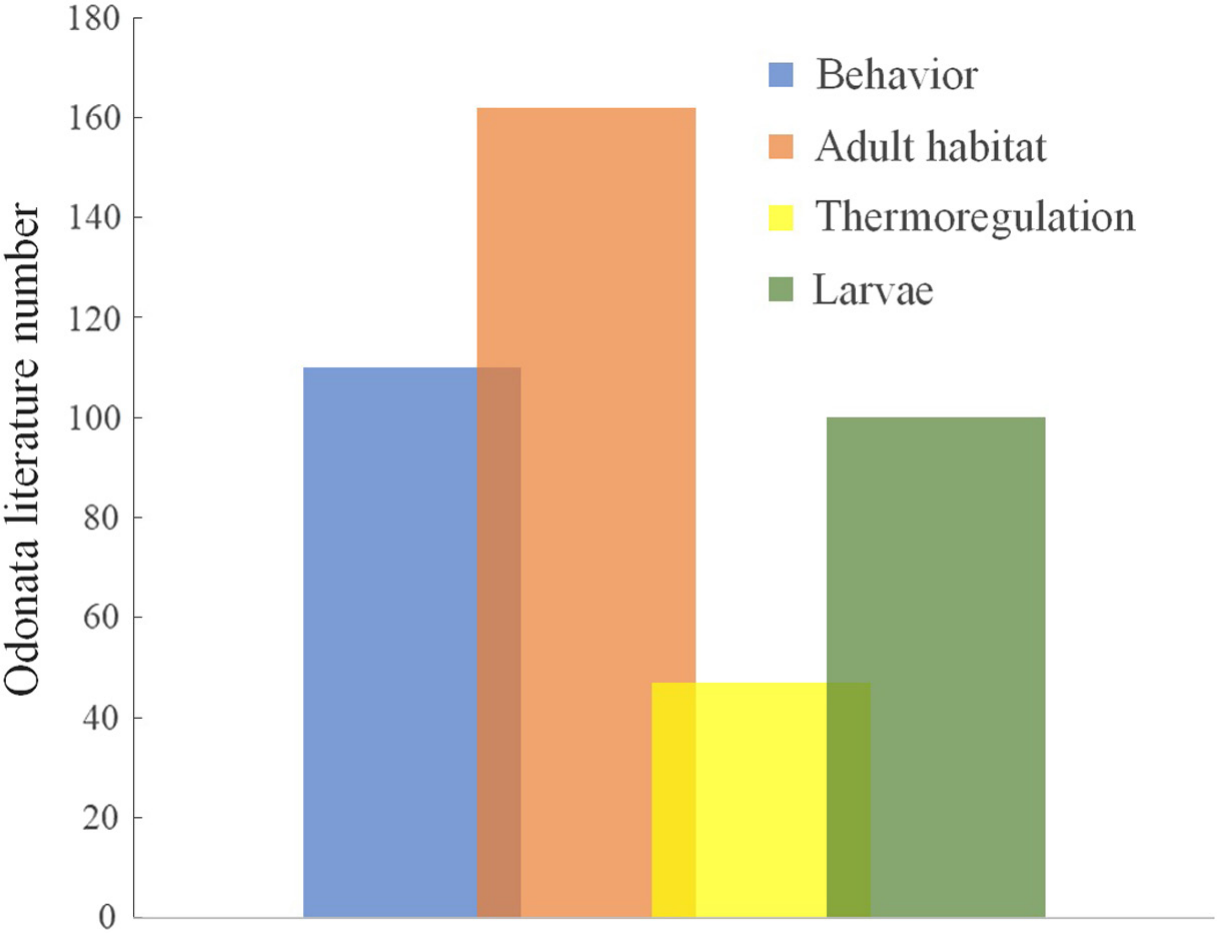
Amount of literature containing records of habitats that Odonata species typical of the Amazon inhabit (detailed in Appendix 1—AMO‐TB available: Dryad repository).

The category of traits with the most available literature referred to the habitat preference of adults. The types of environments colonized by Amazonian species were reported in 162 references (Figure 13). The second‐best studied category was reproductive behavior, recorded in 110 consulted references. Third, we have the category of larval habitats and habits, with a total of 100 references. Finally, thermoregulation was the most underrated category of traits, containing only 47 references (for more details, see Appendix 2 and Appendix 9).

## 4 DISCUSSION

### 4.1 Morphometrics

Obtaining morphometric data are very laborious, mainly because large numbers of specimens are needed, as populations of the same species in different environments may exhibit high phenotypic plasticity (Bowman et al., 2018) or vary along geographic (Johansson et al., 2006) and anthropogenic gradients (Sukhodolskaya, 2013). For insects, the situation is even more complex due to the delicate and diminutive nature of their body structures. Additionally, the availability of specimens in museums and scientific collections is also decisive for the success of obtaining this type of data, which in turn, can present unwanted noise due to possible taxonomic identification errors. Thus, the importance of discussions that seek to identify key morphometries is evident. Hence, this kind of study provides a set of morphological variables from a single trace, greatly facilitating the advancement in fields like ecology and evolution.

The literature indicates that morphometrics can be transformed into indices and ratios that, in turn, give rise to insights that only raw values could not provide. Some of these indices are already relatively well–studied and known to science, such as Wing Load and Wing Stroke, both linked to flight performance and dispersion (Hall & Willmott, 2000; McCauley, 2013; Resende et al., 2021; Wootton, 2020) and widely used in the aeronautical industry (Liang et al., 2014).

Studies show that body size is often evaluated by rival males at the time of dispute or even by potential reproductive partners and can therefore play a role in sexual selection (Suhonen et al., 2008). Biomass, which is linked to body size, reflects physiological issues, such as the level of energy reserves and immunocompetence (Contreras‐Garduño et al., 2006; Vilela, Del‐Claro, et al., 2017). Additionally, there is strong evidence that total body size is positively correlated with male dragonfly flight ability and agility (Vilela, Del‐Claro, et al., 2017). Dutra and De Marco et al. (2015) demonstrate that body size is also a determinant trait for habitat selection in Odonata and varies between suborders. According to Misof (2002), there is a positive relationship between the size and speciation rate of dragonflies. Thus, this characteristic is very important to consider when addressing the functionality of the species.

*Mecistogaster* had the longest body length, as its name implies (from the Greek, “mecister = long” and “gaster = abdomen”). This genus has a striking feature: its long abdomen. Together with slender and long wings, these species exhibit slow and non‐linear flight, but with special foraging abilities, since they are able to steal spiders' prey directly from their webs (Gorb, 2019). Dragonflies classified as fliers and belonging to the endothermic group stood out in terms of thorax size, wing width, and head. As they are constantly in flight, these insects have well‐developed wing muscles, reflecting the larger size of the thorax. Moreover, the greater width of the wings allows them to be gliders and have longer flight times (Corbet & May, 2008; May, 1991).

The leg segments also seem to be an important trait to separate groups, as occurred with representatives of the Calopterygidae and Dicteriadidae. For a dragonfly as an adult and therefore winged, legs lack locomotor function but are often used to grip surfaces during rest, as well as capture and handle prey during flight (Garrison et al., 2006). The legs also play a fundamental role in reproduction, as males of several species, especially damselflies (more details on the topic of Reproductive behavior), use them when attaching their appendages to the female's pronotum during copulation, a movement called tandem. The opposite may also apply, as females have been observed to avoid mating attempts by harassing males using leg movements (Ruppell, 1989). However, we emphasize that the leg parts were not analyzed from a sexual selection point of view.

The genus *Perithemis* was one of the smallest Anisoptera, mainly concerning body size and abdomen. Species of this genus are typical of lentic environments and with ample sunlight. Therefore, small bodies prove to be an advantage, since the high surface/volume ratio of their bodies would facilitate constant exchanges of heat with the environment, thus avoiding possible overheating (De Marco et al., 2015).

Data dispersion is widely used as a metric in several types of research, as it can provide different insights from those obtained from measures of central tendency (Gotelli & Ellison, 2011). It was evident that total body size and abdomen length vary greatly within both suborders when observing the standard deviation of morphometric traits. However, in an overview, there are similarities between the patterns of deviation between the suborders. The greater morphometric variation by the suborder Anisoptera may be linked to taxonomic limitations, since these genera (e.g., *Erythrodiplax*) present great debates regarding their true taxonomic classification (Borror, 1942; Neiss et al., 2018). Therefore, represented by species of different shapes and sizes within a genus that may not necessarily belong to the same evolutionary lineage (Bastos et al., 2021). Intuitively, another factor that could explain this pattern of greater variation within certain genera would be the number of species that compose them, since the greater morphometric variation occurred preferentially in species of very diverse genera.

### 4.2 Types of behavior

Odonata has a highly complex reproductive behavior, secondary male genitalia, complex courtship behaviors, a territorial dispute between males, and different types of oviposition, among other remarkable behaviors (Corbet, 1999). One of the most notorious is the aggressive and territorial behavior of many species. Territorial males defend areas, which can vary from a few centimeters to a few square meters, usually with resources considered valuable for females to oviposit (Suhonen et al., 2008). It is evident that territorial males increase access to females by defending a fixed territory. On the other hand, males spend more energy during agonistic interactions and become more vulnerable due to their constant patrols and conspicuous displays (Suhonen et al., 2008). However, it is worth highlighting the advantages found by some species and even individuals of the same population in not protecting a fixed territory, assuming different strategies to access females, such as satellite non‐territorial males that actively search or wait for females on the oviposition sites or surrounding vegetation (Fincke, 1985).

Agonistic displays during disputes are communicative, that is, they convey visual messages to their co‐specifics (Gonzalez‐Santoyo et al., 2014; Vilela, Tosta, et al., 2017). Dominant males are easily observed patrolling their territories and often resolve disputes without any contact, using some type of display, which can range from specific wing movements to short chase flights. This type of behavior has evolved within several groups of animals due to the fitness acquired by males who, instead of battling to the death, resolve their disputes without greater energy demands (Guillermo‐Ferreira et al., 2015).

Once females are inseminated (whether they have been courted or not), the oviposition begins, which can assume different behaviors according to the species: guarding with contact or without contact, or even without guarding at all (Corbet, 1999). According to our research, most Odonata species exhibit tandem behavior and non‐contact guarding. Additionally, some species overlap different traits within this category. These dualities can be explained by the intrinsic phenomena of the group, such as high behavioral plasticity and the inherent costs of mate‐guarding by males (Helebrandová et al., 2019; Resende et al., 2021).

Species reproductive strategies during oviposition in Zygoptera are mostly endophytic, while Anisoptera usually performs exophytic oviposition. These differences can be explained by the morphological differences of the suborders. In general, damselflies have ovipositors specialized in perforating living (as is the case of most Coenagrionidae) or decomposing (Polythoridae) plant tissues (Bentes et al., 2014; Rodrigues et al., 2019). This type of oviposition requires an ovipositor with the presence of small teeth on the sides of the vulva and a spine‐shaped structure called a stylet (Matushkina & Gorb, 2007). On the contrary, most dragonflies (except for aeshnids) present ovipositors adapted to oviposition on the water surface, with a reduced ovipositor appendage, anchored to a strong and complex muscular system specialized in the contraction of the entire ovipositor apparatus and abdomen, which aims to expel the egg mass when the female touches the tip of her abdomen into the water (Matushkina, 2011).

### 4.3 Adult habitat preference

Dragonflies are insects with high dispersal capacity, being found in all types of aquatic environments. Among the eight environment types, the highest occurrence of restricted species was in stream environments. This type of environment varies from shallow channels located in the thalweg of geological depressions, generally with little current, bed rich in rocky minerals, and abundant inflow of allochthonous material, to larger volumes of water flowing over wide channels, presenting turbulent waters, sedimented substrate, and greater input of sunlight (Allan & Castillo, 2007). The greater relationship of members of the family Pseudostigmatidae to forest environments is related to the habit of larvae that develop in natural water reservoirs found in fruits (e.g., Brazil nut urchin), bracts of fallen palm trees (Neiss, 2012), and tree trunk holes and inside bamboo internodes (Fincke, 1984). Individuals in this family only move to the water channel when foraging and/or searching for reproductive partners.

Open fields are environments that can range from preserved, such as those found in the ferruginous fields of the Amazon, swampland, and the Cerrado savannas; or impacted by human intervention, pastures, and monocultures. In these types of environments, species of Anisoptera were almost unanimously found, probably due to their size and thermoregulatory strategies (De Marco et al., 2015; Oliveira‐Junior & Juen, 2019), with the only exception of the small zygopteran *I. capreolus*, common in very sunny places.

### 4.4 Thermoregulation

Ectothermic dragonflies depend on an ideal temperature range to carry out their daily activities (May, 1991). Individuals can range from thermal conformers, making convective heat exchanges with the environment and keeping their chest temperature close to air temperature, to heliothermics, requiring direct solar radiation on the body, and can maintain their temperature above air temperature (Shelly, 1982). Species with large body sizes such as Aeshnidae are considered endothermic, as they regulate their temperature through flight, determining heat production (May, 1991).

According to ecophysiological strategies, adopted flight mode (Bomphrey et al., 2016), behavioral changes (May, 1991), habitat selection (Shelly, 1982), or body size (Castillo‐Pérez, May, et al., 2022) affect heat production/maintenance. As for the flight mode, dragonflies can be classified dichotomously into: perchers, individuals that spend most of their time perched and often depend on constant heat exchange with the environment to reach the ideal temperature, thus being categorized as ectotherms; and fliers, individuals that sustain long flights, are generally dragonflies that produce and manage internal heat, considered endothermic. This sustained dichotomy further suggests that there may be a continuum within each of them based on body size (Corbet & May, 2008).

Odonates have many specificities and it is important to evaluate them among the suborders since previous studies show that they have differences in body temperatures (Castillo‐Pérez, Suárez‐Tovar, et al., 2022) and in the type of environment they select, affecting their life history and distribution (De Marco et al., 2015, Oliveira‐Junior & Juen, 2019). Microhabitat selection can be predicted based on knowledge of the thermoregulatory abilities that dragonflies exhibit (De Marco, 1998). Thus, it is essential to survey this bionomic aspect of these species to solve urgent issues associated with the conservation of aquatic systems in the Amazon region.

### 4.5 Geographic distribution

The vast majority of Odonata species occurring in the Amazon are not necessarily restricted to the region, and may, in some cases, be distributed throughout the American continent. Since significant ecological and climatic differences arise across biomes, the range of zones dominated by a species can provide us with information linked to dispersal capacity and environmental tolerance (Renner et al., 2019). In some cases, this distribution pattern may be related to morphological traits of these species, such as body size, or even the shape, size, and venation of the wings (Hefler et al., 2018; Wootton, 2020). In fact, morphology has been considered an important element to understand the biological mechanisms of the group. Furthermore, the spatial distribution of species can be explained by possible relationships with environmental conditions that define the type of environment in which organisms live (Vandermeer, 1972). The answers found in this study may also reflect the distribution of larvae since several studies point to a strong congruence between larvae and adults of Odonata (Mendes et al., 2017; Valente‐Neto et al., 2016). However, there are still many questions to be explored, as it is not yet known for sure whether the body size of odonates is filtered by the current ecological conditions or is an evolutionary factor that shaped the distribution of organisms until the current days.

### 4.6 Larval habits and habitats

The order Odonata presents very marked differences between the larvae of the suborders Anisoptera and Zygoptera, and these characteristics are due to their specialization in the aquatic environments where they establish, aiming to guarantee their survival (Corbet, 1999). Here, we demonstrate that most Zygoptera genera studied occur exclusively in lotic environments. This must be related to higher oxygen concentrations in fast‐flowing waters, because depending on the type of respiration that this group presents, a greater amount of oxygen is needed in the water through the caudal lamellae (Ramirez, 2010). However, for Anisoptera, we showed that most genera occur in both lentic and lotic environments. This group is considered more generalist when compared to Zygoptera, and due to this greater niche breadth, they can occur in habitats with different water flows (Oliveira‐Junior & Juen, 2019).

Considering the micro‐habitats in which the larvae occur, most Zygoptera genera are predominant in places with inorganic sediments (sand, gravel), with the presence of leaf banks and roots. This preference for these types of substrates is related to the life habits, such as prey capturing and sheltering. Within the Zygoptera, we showed that the larvae have mainly climbing and clasping habits, which justifies their preference for root substrates. In addition, the leaf banks serve as a shelter for the larvae and, due to the large presence of prey in this substrate, it allows for the capture of food (Carvalho & Nessimian, 1998). For Anisoptera, the larvae occur mainly in organic sediments, fragments of trunks, branches, and in places with rocks and leaf banks. This pattern is also related to the habit of this group, as most genera are sprawlers, that is, moving over and through the leafy banks and plant fragments, and burrowers, which live buried within inorganic sediments, such as gravel and sand (Assis et al., 2004).

The variety of habits that the larvae present can be explained by the great variation in morphological characteristics, as these characteristics can express their importance and functionality within the community and in their micro‐habitats (Mendes et al., 2020). Zygoptera that were predominantly graspers and climbers generally have a cylindrical body and more elongated legs (e.g., *Dicterias*, *Mnesarete*, and *Heliocharis*), which allow them to attach to vegetation (Carvalho & Nessimian, 1998). However, we saw that *Argia* and *Heteragrion*, despite having the clasping behavior, are also sprawlers and we attribute this to the dorsoventrally flattened body shape and shorter legs in these genera (Novelo‐Gutiérrez, 1992). About the swimming habit, little represented in our work, only by the larvae of *Mecistogaster*, *Microstigma*, and *Perilestes*, which generally have a more elongated abdomen, which is laterally flattened, with the presence of thin legs and long leafy lamellae. For Anisoptera, most genera are sprawlers, especially those belonging to the Libellulidae and Corduliidae families, or burrowers, as described for Gomphidae. To stay buried in the substrate, the larvae have a more elongated, fusiform body and the presence of short fossorial legs, and specialized structures, such as projections on the posterior portions of the tibia. In addition, they may also present specialized modifications that facilitate their breathing when they are buried, such as the presence of prolongation in the last segment of the abdomen, as in the larvae of *Phyllocycla* and *Aphylla* (Assis et al., 2004). In addition, we also found within this suborder clasping organisms, mainly in Aeshnidae, which were shown in our ordination to be strongly associated with the root substrate, or some exceptions within the Libellulidae family, such as the genus *Tholymis*, which was exclusively clasper.

### 4.7 Gaps in the literature

The three categories of traits with more literature, respectively, were: habitat preference of adults, reproductive behavior, and habitat/habit of the larvae. The types of environments colonized by Amazonian species were reported in 162 bibliographies (for more, see Appendix 1 and Appendix 9 in repository Dryad). We attribute this result to the abundance of checklists available, given that faunal surveys are essential for any type of research and/or animal management. However, here we emphasize that larval habits need more attention since only five articles were found directly dealing with this subject. Finally, despite thermoregulation being widely debated in several ecological studies with Odonata (Batista et al., 2021; De Marco et al., 2015), determining the thermoregulatory strategy of a species is not a simple task. There is a need for special equipment and techniques for this, including field observation to measure the body temperature of individuals, also of behaviors that are linked to certain strategies, for example, displays that help control temperature (e.g., obelisk posture), flight duration or micro‐habitat preference (De Marco, 1998). Devices that measure temperature with high precision and that are adapted to be quick in their measurements, as small organisms quickly change their temperature when handled and this leads to technical misconduct. However, as it is one of the main arguments to explain the distribution of species in ecological works, it is urgent to develop studies so that we can increase knowledge on the subject. Another starting point is also through physiological and anatomical analyses, since dragonflies have specific structures that determine their way of reaching their ideal temperature, as is the case of hemolymph transport systems (endothermic) or surface/volume ratio of species body (Guillermo‐Ferreira & Gorb, 2021; Rocha‐Ortega et al., 2020).

## 5 FINAL CONSIDERATIONS

Our research efforts have gathered information on the basic biology of several species of Odonata found in the Amazon. However, we highlight the lack of data in several analyzed categories, mainly referring to thermoregulation and larval habits. Additionally, we suggest as a matter of urgency, studies that delimit a consensus in the definition of terms frequently used within the literature, as is the case of the divergent characterization of the types of oviposition. A correlation between certain functional traits was evident, especially those that express complementary information. Given the difficulty in compiling and collecting this type of data, we recommend a selection of traits based on the information presented here when building future functional matrices. Finally, we suggest research to evaluate how functional traits are influenced by environmental factors or phylogenetic relationships. Additionally, since we provide the traits and their respective literature, another possibility would be to perform sciences evaluating the quality and amount of information available, for example, by functional groups or even taxonomic groups. In addition, decision‐makers can use this Trait Bank to identify functionality or infer ecosystem services that should be prioritized in different environmental scenarios and conservation strategies for Odonata species, especially in the Amazon region.

## AUTHOR CONTRIBUTIONS

**Victor Rennan Santos Ferreira:** Conceptualization (equal); data curation (equal); formal analysis (equal); investigation (equal); methodology (equal); project administration (equal); software (equal); supervision (equal); validation (equal); writing – original draft (equal); writing – review and editing (equal). **Bethânia Oliveira de Resende:** Conceptualization (equal); data curation (lead); supervision (lead); writing – review and editing (equal). **Rafael Costa Bastos:** Formal analysis (lead); methodology (lead); software (lead); writing – review and editing (equal). **Joás Silva da Brito:** Data curation (equal); methodology (equal); writing – review and editing (equal). **Fernando Geraldo de Carvalho:** Data curation (equal); formal analysis (equal); investigation (equal); writing – review and editing (equal). **Lenize Batista Calvão:** Data curation (equal); investigation (equal); validation (equal); writing – review and editing (lead). **José Max Barbosa Oliveira‐Junior:** Data curation (equal); funding acquisition (equal); validation (equal); writing – review and editing (equal). **Ulisses Gaspar Neiss:** Data curation (equal); validation (equal); writing – review and editing (equal). **Rhainer Ferreira:** Conceptualization (equal); investigation (equal); supervision (equal); validation (equal); writing – review and editing (equal). **Leandro Juen:** Conceptualization (equal); data curation (equal); funding acquisition (equal); investigation (equal); resources (equal); supervision (equal); writing – original draft (equal); writing – review and editing (equal).

## ACKNOWLEDGEMENTS

We are grateful to the CAPES, CNPq and FADESP for subsidizing research grants. We also acknowledge the UFPA, LABECO and PPGECO for providing the physical and intellectual structure. Finally, we thank Dr. Diogo Vilela and other researchers for their contributions.

## CONFLICT OF INTEREST STATEMENT

All authors declare that there is no conflict of interest in this research.

### OPEN RESEARCH BADGES

This article has earned Open Data and Open Materials badges. Data and materials are available at [https://doi.org/10.5061/dryad.brv15dvdg].

## Data Availability

The AMO‐TB and the data that support the findings of this study are available at: Dryad ‐ https://doi.org/10.5061/dryad.brv15dvdg.
